# Measuring the Success of HIV-1 Cure Strategies

**DOI:** 10.3389/fcimb.2020.00134

**Published:** 2020-04-07

**Authors:** Jordan Thomas, Alessandra Ruggiero, William A. Paxton, Georgios Pollakis

**Affiliations:** ^1^Department of Clinical Infection, Microbiology and Immunology, Institute of Infection and Global Health, University of Liverpool, Liverpool, United Kingdom; ^2^Immune and Infectious Disease Division, Academic Department of Pediatrics (DPUO), Bambino Gesù Children's Hospital, Rome, Italy

**Keywords:** HIV-1, persistence, latency, latent reservoir, DNA/RNA quantification

## Abstract

HIV-1 eradication strategies aim to achieve viral remission in the absence of antiretroviral therapy (ART). The development of an HIV-1 cure remains challenging due to the latent reservoir (LR): long-lived CD4 T cells that harbor transcriptionally silent HIV-1 provirus. The LR is stable despite years of suppressive ART and is the source of rebound viremia following therapy interruption. Cure strategies such as “shock and kill” aim to eliminate or reduce the LR by reversing latency, exposing the infected cells to clearance via the immune response or the viral cytopathic effect. Alternative strategies include therapeutic vaccination, which aims to prime the immune response to facilitate control of the virus in the absence of ART. Despite promising advances, these strategies have been unable to significantly reduce the LR or increase the time to viral rebound but have provided invaluable insight in the field of HIV-1 eradication. The development and assessment of an HIV-1 cure requires robust assays that can measure the LR with sufficient sensitivity to detect changes that may occur following treatment. The viral outgrowth assay (VOA) is considered the gold standard method for LR quantification due to its ability to distinguish intact and defective provirus. However, the VOA is time consuming and resource intensive, therefore several alternative assays have been developed to bridge the gap between practicality and accuracy. Whilst a cure for HIV-1 infection remains elusive, recent advances in our understanding of the LR and methods for its eradication have offered renewed hope regarding achieving ART free viral remission.

## Introduction

Infection with human immunodeficiency virus type-1 (HIV-1) requires life-long adherence to antiretroviral therapy (ART) due to the presence of latently infected cells that are central to viral persistence and rebound viremia following ART interruption (Chun et al., 1997, 1998, 1999; Finzi et al., 1997; Perelson et al., 1997; Wong et al., 1997; Davey et al., 1999; Rosenbloom et al., 2017). HIV-1 primarily infects activated CD4 T cells, where genomic RNA is reverse transcribed into DNA and stably integrated into the host genome. Integrated proviral DNA therein serves as the template for HIV-1 gene expression and genomic RNA production, driven by T cell activation induced transcription factors such as NF-κB (Liu et al., 1992; Kinoshita et al., 1998). The latent reservoir (LR) is established when a small subset of activated CD4 T cells, harboring proviral DNA, revert to a resting memory phenotype with reduced gene expression, rendering the cell non-permissive for HIV-1 production but providing a sanctuary to evade the immune response and ART (Hermankova et al., 2003; Siliciano and Greene, 2011). The LR is stable over long periods in therapy suppressed individuals; the result of infection in naturally long-lived memory CD4 T cells that are continually replenished by clonal expansion and homeostatic proliferation (Finzi et al., 1999; Siliciano et al., 2003; Bailey et al., 2006; Chomont et al., 2009; Maldarelli et al., 2014; Wagner et al., 2014; Cohn et al., 2015; Lorenzi et al., 2016; Simonetti et al., 2016; Hosmane et al., 2017). Latently infected cells therefore represent the principle barrier to an HIV-1 cure and should be specifically targeted by novel treatment and eradication strategies.

To date, an effective cure for HIV-1 infection has been achieved twice via CCR5Δ32/Δ32 hematopoietic stem cell transplantation and in both cases latently infected cells were eliminated and replaced with HIV-1 resistant donor cells (Hutter et al., 2009; Gupta et al., 2019). Whilst this method is not feasible for widespread use, its repeated success proves the principle that HIV-1 cure strategies must either eliminate (sterilizing cure) or silence (functional cure) the LR. Proposed cure strategies such as “shock and kill” aim to eliminate the LR by utilizing latency reversing agents (LRAs) during ART mediated virus suppression to drive expression of HIV-1 from latently infected cells, exposing those cells to viral cytopathic effects or immune clearance whilst limiting *de novo* infections (Deeks, 2012). An alternative and conceptually opposing method, “block and lock,” aims to reinforce viral latency and therefore maintain the provirus in an inactivate state in the absence of ART (Mousseau et al., 2015; Méndez et al., 2018). Additionally, therapeutic vaccination based approaches aim to silence the LR by inducing strong HIV-1 specific T cell responses to aid immune control of the infection following ART cessation (Mylvaganam et al., 2015; Pantaleo and Levy, 2016).

Measuring the success of HIV-1 cure and vaccine strategies requires highly sensitive and accurate assays and there is currently no consensus as to the most appropriate method to utilize. Several technical challenges limit the ability to measure accurately the size of the LR, including the paucity of cells infected with replication competent provirus and the vast heterogeneity of the HIV-1 genome. Culture based assays such as the viral outgrowth assay (VOA) are routinely used to measure the LR but are labor and resource intensive and invariably underestimate the size of the replication competent reservoir (Ho et al., 2013; Bruner et al., 2015). Conversely, PCR based assays offer a more practical approach to proviral quantification but overestimate the size of the LR by indiscriminately measuring defective viral genomes that predominate the *in vivo* landscape (Ho et al., 2013).

Despite the success of ART in reducing HIV-1 associated mortality, the global burden of the disease necessitates the urgent development of a cure or vaccine and both understanding and accurately measuring the LR is crucial in the path toward HIV-1 eradication. In this review, we will focus on the mechanisms that facilitate the establishment and maintenance of the HIV-1 LR, some of the prominent methods proposed to achieve a cure and the developments and challenges on the way to measuring their success.

## The Latent Reservoir

### Establishing Latency

The HIV-1 LR can be defined as the fraction of cells harboring transcriptionally silent proviral DNA that are capable of producing infectious virions following activation (Eisele and Siliciano, 2012). Resting memory CD4 T cells are the primary host of the LR but HIV-1 infection in these cells is inefficient due their low co-receptor expression and inherent restrictions to reverse transcription (Pierson et al., 2000; Baldauf et al., 2012). Nevertheless, there is evidence that HIV-1 can infect resting CD4 T cells directly or via cell-to-cell transmission, though infection in these cells is associated with slower replication kinetics (Swiggard et al., 2004, 2005; Agosto et al., 2007, 2018; Plesa et al., 2007; Vatakis et al., 2007; Lassen et al., 2012). Alternatively, latency is established when a subset of infected, activated CD4 T cells revert to a resting memory phenotype, effectively silencing viral gene expression whilst sustaining the proviral DNA long-term (Chun et al., 1995). The provirus is maintained in a quiescent state in these cells via host factors such as epigenetic suppression, depletion of transcription factors such as NF-κB and transcriptional interference due to integration into expressed genes, reviewed in more detail (Cary et al., 2016).

Amongst the pool of viral genomes integrated into host cells, only a small fraction are replication competent and therefore capable of producing infectious HIV-1 virions following T cell activation (Sanchez et al., 1997; Ho et al., 2013; Bruner et al., 2016; Imamichia et al., 2016). Instead, the majority of the reservoir exists as defective provirus, unable to support HIV-1 infection due to deletions, insertions and hypermutation introduced into the genome during reverse transcription (Ho et al., 2013; Bruner et al., 2016). Despite this, viral rebound from the LR following ART cessation is rapid, leading to detectable viremia within weeks of therapy interruption (Chun et al., 1999; Davey et al., 1999). Additionally, initiating ART early in infection is not sufficient to stop the formation of the LR, suggesting the LR is established and disseminated early (Chun et al., 1998; Whitney et al., 2014; Colby et al., 2018), even in vertically infected children that started ART soon after birth (Persaud et al., 2013; Ananworanich and Robb, 2014; Giacomet et al., 2014; Tagarro et al., 2018).

### Maintaining the Reservoir

The half-life of the LR is estimated to be 3.6 years in patients with sustained viral suppression, meaning that eradication of the LR is not possible within a lifetime and adherence to ART must therefore be lifelong (Siliciano et al., 2003; Crooks et al., 2015). The natural longevity of memory T cells contributes to the persistence of the LR, however, its long-term stability indicates that this pool of cells is continually replenished notwithstanding effective ART. Two mechanisms have been proposed as drivers of LR maintenance: ongoing virus replication in anatomical compartments with sub-optimal drug concentrations and/or clonal expansion of latently infected cells (Sengupta and Siliciano, 2018). Ongoing replication of HIV-1 would lead to the accumulation of genetically diverse HIV-1 provirus, integrated into various positions of the host genome, therefore, researchers have monitored viral evolution and integration sites in ART suppressed patients to determine the mechanism of LR propagation. Separate studies have demonstrated a high proportion of genetically indistinct viral genomes, as well as identical integration sites recovered from different cells, indicating that these cells must arise from proliferation as appose to subsequent HIV-1 replication (Josefsson et al., 2013; Wagner et al., 2013, 2014; Maldarelli et al., 2014; von Stockenstrom et al., 2015; Wang Z. et al., 2018). Indeed, memory T cells are maintained by homeostatic proliferation in response to IL-7, and several studies have shown that this process drives LR persistence without inducing HIV-1 gene expression (Agosto et al., 2007; Chomont et al., 2009; Archin et al., 2012). These studies, however, do not demonstrate that the expanded viral clones are replication competent and therefore, their contribution to HIV-1 persistence is unclear. One study, in fact, revealed that of a population of 75 expanded clones, none of the proviral sequences were found to be replication competent (Cohn et al., 2015). To address this, researchers have utilized full-length sequencing approaches to demonstrate that within the replication competent proviral pool, 55–60% of viral genomes had identical sequences in different cells (Lorenzi et al., 2016; Hosmane et al., 2017). Further, a recent longitudinal analysis revealed that rebound viremia matched archival provirus that was present prior to ART initiation and during long term ART suppression (De Scheerder et al., 2019). Taken together, these studies indicate that cellular expansions play a key role in the maintenance of the replication competent viral reservoir in long-term therapy suppressed patients, providing a clear mechanism for HIV-1 persistence and a source of rebound viremia following ART cessation.

On the other hand, the contribution of low-level virus replication in anatomical compartments with sub-optimal drug concentrations, such as lymph nodes (LN), to LR maintenance is a topic of continued debate (Fletcher et al., 2014; Fukazawa et al., 2015; Lorenzo-Redondo et al., 2016; Nolan et al., 2017; Bozzi et al., 2019). Generally, most studies demonstrate little evidence of provirus evolution in ART suppressed patients, refuting the likelihood that ongoing replication is continually seeding the reservoir (Bailey et al., 2006; Chomont et al., 2009; Josefsson et al., 2013; Hiener et al., 2017; Lee et al., 2017; Van Zyl et al., 2017; Bozzi et al., 2019; De Scheerder et al., 2019). Additionally, ART intensification studies have been unable to reduce low-level viremia, suggesting that this phenomena is a result of stochastic activation of latently infected cells, rather than continued rounds of replication (Dinoso et al., 2009; McMahon et al., 2010; Anderson et al., 2011; Gandhi et al., 2012). Nevertheless, evidence from various studies has supported the hypothesis that ongoing replication takes place notwithstanding suppressive ART. Intensification of the integrase inhibitor raltegravir, for example, led to transient increases in 2-LTR circular DNA which, as products of failed integration events, suggests inhibition of new infections (Buzón et al., 2010; Hatano et al., 2013; Puertas et al., 2018). Further, evidence of virus evolution within the LN of therapy suppressed patients was also suggested as an indication of ongoing replication (Lorenzo-Redondo et al., 2016). However, two groups have reported that this is instead an artifact of rapidly decaying viral species associated with early antiretroviral treatment (Kearney et al., 2017; Rosenbloom et al., 2017).

### The Hosts of the Reservoir

Critical to the elimination of HIV-1 is the elucidation of the specific anatomical and cellular reservoirs of HIV-1. Various differentiation states of CD4 T cells appear to play important roles in the establishment and maintenance of the LR as well as viral recrudescence following ART interruption (Buzon et al., 2014; Kulpa and Chomont, 2015; Banga et al., 2016; De Scheerder et al., 2019; Falcinelli et al., 2019). As discussed above, the LR is primarily hosted in memory CD4 T cells, specifically, central (T_CM_), transitional (T_TM_), effector memory (T_EM_), and memory stem (T_SCM_) cells, although the exact contribution of each cell type to the replication competent reservoir is still to be determined (Chomont et al., 2009; Buzon et al., 2014; Soriano-Sarabia et al., 2014; Banga et al., 2016, 2018; Kwon et al., 2020). Recently, CD32+ CD4 T cells have been proposed to be a major host of the LR, whereby selection of this cell population resulted in significant enrichment of inducible provirus (Descours et al., 2017; Darcis et al., 2020). Conflicting reports, however, have failed to replicate this finding and the contribution of CD32+ CD4 T cells to HIV-1 persistence and rebound remains controversial (Abdel-Mohsen et al., 2018; Badia et al., 2018; Bertagnolli et al., 2018; Martin et al., 2018; Osuna et al., 2018; Pérez et al., 2018). Nonetheless, the use of CD32 as a marker of latent infection is a topic of particular interest and may provide a mechanism by which the LR can be specifically targeted.

As well as categorizing cells based on their differentiation state, these cells can also be subdivided based on their functional properties. Accordingly, specific CD4 functional sub-sets, such as regulatory T cells (T_reg_), Th17 cells and follicular T helper cells (T_fh_) are now being characterized in more detail to determine which cells are the primary contributor to HIV-1 latency. T_reg_ cells modulate the immune response through regulation of T cell proliferation and differentiation whilst Th17 cells are critical to maintaining mucosal immunity via secretion of IL-17 and the balance of these two cell subsets is therefore critical in providing effective immune function (Valverde-Villegas et al., 2015). Both T_reg_ and Th17 cells have been shown to harbor a high proportion of the LR in therapy suppressed patients and as such, may be an important target in HIV-1 cure efforts (Tran et al., 2008; Alvarez et al., 2013; Sun et al., 2015; Christensen-Quick et al., 2016; Caruso et al., 2019).

Due to the inherent difficulty of sampling from tissues, most LR studies are based on the analysis of peripheral blood. In recent years, more research has focused on studying anatomical reservoirs such as lymph nodes (LN) and gut associated lymphoid tissue (GALT), as these sites are enriched in activated CD4 T cells (Chun et al., 2008; Di Mascio et al., 2009; Yukl et al., 2010; Churchill et al., 2016). Follicular T helper cells (T_fh_), resident within the B cell follicle of LN have recently been identified as a major host of the replication competent viral reservoir (Buzon et al., 2014; Banga et al., 2016, 2019). These studies demonstrate the importance of individual anatomical and cellular hosts of the LR to HIV-1 persistence and highlight that HIV-1 eradication studies will need to not only target these sites, but also efficiently and specifically measure the LR within these compartments.

In addition to lymphocytes, a number of other cells types such as macrophages and plasmacytoid dendritic cells (pDCs) are potential hosts of the LR, and despite being infected at a lower frequency, may play an important role in viral persistence (Centlivre et al., 2011). Studies suggest that macrophages infected with HIV-1 are resistant to cell mediated immune clearance as well as virus induced cell death and may therefore represent a significant hurdle to cure (Swingler et al., 2007; Clayton et al., 2018). Further, replication competent provirus has recently been recovered from macrophages in long-term ART suppressed patients, indicating that cure strategies targeting only lymphocytes may not be sufficient (Ganor et al., 2019). Infection of macrophages with HIV-1 can facilitate entry of virus into anatomical sanctuary sites such as the brain and central nervous system (CNS), therefore providing an additional barrier to HIV-1 eradication (Castellano et al., 2017; Wong et al., 2019).

## HIV-1 Cure Strategies

### Progress Toward a Cure

The progress toward the development of a functional or sterilizing cure for HIV-1 has been significantly hindered by the presence of the LR. Currently, two people have been cured of HIV-1 infection, the so-called Berlin and London patients, who since receiving allogenic stem cell transplantations from CCR5Δ32/Δ32 donors, have consistently tested negative for viral rebound for over 10 and 2 years, respectively, without ART (Hutter et al., 2009; Gupta et al., 2019). In these cases, the infected cell pool was significantly depleted during pre-transplant conditioning and replaced with donor cells that are resistant to infection with R5-tropic virus due a large deletion in the CCR5 co-receptor (Liu et al., 1996). Due to the relative paucity of CCR5Δ32/Δ32 donors and the unique circumstances predetermining these cases, this type of cure is not feasible for widespread use, it does however emphasize the basic principle of HIV-1 cure; silence or eradicate the HIV-1 LR.

### Shock and Kill

**Figure 1:**
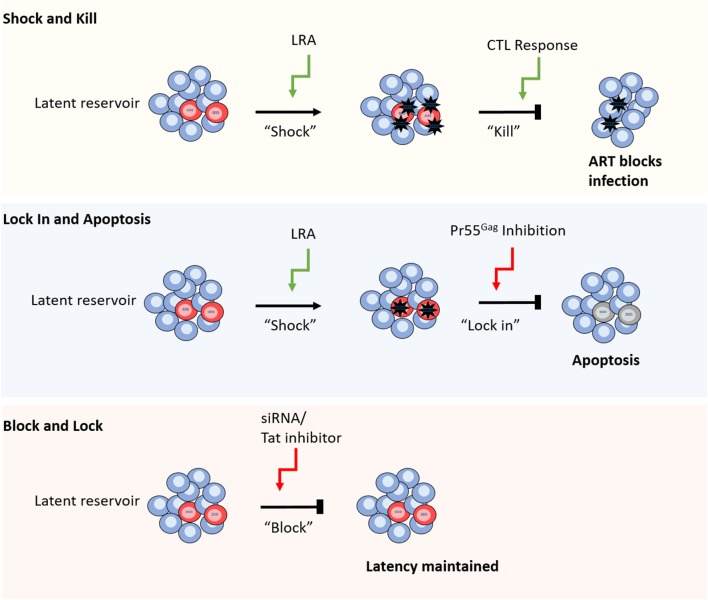
Different strategies for HIV-1 cure. From top to bottom. Shock and kill relies on reversal of latency using a range of different compounds including TLR agonists and HDACis, followed by CTL mediated cell clearance, whilst ART blocks new infections caused by virus release. Lock in and apoptosis utilizes latency reversal agents, as well as a Pr55Gag inhibitor to block virus budding from the cell. The build-up of viral RNA and proteins leads to apoptosis of the infected cell. Block and lock approaches aim to reinforce latency mechanisms by using siRNAs or Tat inhibitors to disrupt cellular epigenetic regulators or viral replication, respectively (red cells represent HIV-1 latently infected cells).

One of the most prominent approaches to achieve HIV-1 cure is “shock and kill”; the use of latency reversing agents (LRAs) to induce viral gene expression and productive infection in latently infected cells, exposing those cells to immune clearance or the viral cytopathic effect with the aim of reducing the size of the LR and limiting viral rebound (Figure 1; Deeks, 2012). A major challenge in this approach is the ability to achieve broad and efficient latency reversal without eliciting toxic side effects or global immune activation. Early latency reversal studies that utilized interleukin-2 (IL-2) to induce HIV-1 activation produced a toxic “cytokine storm” response and did not sufficiently reduce the size of the LR when the dosage was lowered to safer levels (Prins et al., 1999; Lafeuillade et al., 2001). Instead, novel LRAs induce HIV-1 gene expression either by activating cellular transcription factors, such as NF-κB, or by altering the chromatin structure of the integrated provirus. In their review, Abner and Jordan extensively list published LRAs and categorize them into six groups based on their mechanism of action as follows: histone post-translational modification modulators, non-histone chromatin modulators, NF-κB stimulators, TLR agonists, extracellular stimulators, and a miscellaneous category of unique cellular mechanisms (Abner and Jordan, 2019).

Some of the prominent LRAs currently in use in ongoing clinical trials include histone deacetylate inhibitors (HDACi) and histone methyltransferases inhibitors (HMTi), which induce HIV-1 expression by reversing epigenetic silencing (Lehrman et al., 2005; Agosto et al., 2007; Archin et al., 2012, 2014a; Delagrèverie et al., 2016; Aid et al., 2018; Abner and Jordan, 2019). Alternatively, protein kinase C (PKC) agonists (Williams et al., 2004; Perez et al., 2010; Marsden et al., 2018) and CCR5 agonists (López-Huertas et al., 2017; Madrid-Elena et al., 2018) stimulate latent HIV-1 by activating NF-κB. The use of toll like receptor (TLR) agonists as LRAs has also been explored, as they stimulate immune signaling pathways, leading to HIV-1 expression (Thibault et al., 2009; Novis et al., 2013; Alvarez-Carbonell et al., 2017). As an alternative to conventional LRAs, the use of a polyvalent HIV-1 vaccine has been proposed as a potential candidate to initiate latency reversal, based on the rationale that latently infected CD4 T cells express HIV-1 specific T cell receptors (TCR) and are therefore activated by HIV-1 antigen presentation (Pankrac et al., 2017). These molecules have so far resulted in modest viral activation *in vivo*, however, two recent studies have demonstrated potent and persistent latency reversal in mouse and SIV models in multiple tissues as well as peripheral blood: one utilized a LRA that activates the non-canonical NF-κB pathway (Nixon et al., 2020) and the other combined CD8 T cell depletion with IL-15 stimulation (McBrien et al., 2020). Evidence suggests that the capacity of different LRAs to activate HIV-1 gene expression is varied amongst different CD4 T cell subsets due to the diversity of the mechanisms that drive viral latency across these subsets (Grau-Expósito et al., 2019; Pardons et al., 2019b). Combinations of LRAs could therefore conceivably elicit more global reactivation by acting on different mechanisms that enforce viral latency, and synergy between multiple combinations of LRAs has so far been identified *in vitro* (Darcis et al., 2015; Jiang et al., 2015; Albert et al., 2017; Zaikos et al., 2018; Abner and Jordan, 2019; McBrien et al., 2020; van der Sluis et al., 2020). Nevertheless, achieving global reactivation of HIV-1 from latently infected cells is only part of the challenge; these cells must also be efficiently killed, either by the viral cytopathic effect or by cytotoxic T lymphocyte (CTL) mediated immune clearance. Currently, studies that have achieved latency reversal *in vivo* have failed to reduce the LR or increase the time to viral rebound (Xing et al., 2011; Doyon et al., 2013; Archin et al., 2014a,b, 2017; Elliott et al., 2015), indicating a deficiency in the clearance of infected cells. This impairment of the “kill” response may be due, in part, to loss of HIV-1 specific CTL responses in long-term suppressed patients (Chomont et al., 2018) that may need to be restored in order to achieve sufficient clearance of infected cells (Shan et al., 2012). Importantly, LRAs that activate HIV-1 mRNA expression may not be sufficient to induce the production of viral proteins or infectious virions, and therefore the presentation of viral antigens to CTLs via major histocompatibility complex class 1 (MHC-1) may be limited (Clutton and Jones, 2018; Grau-Expósito et al., 2019) Additionally, treatment with LRAs may specifically inhibit the clearance of infected cells, for example, HDACis have been shown to impair CTL function and the LRA, disulfiram, may induce an anti-apoptotic state that promotes cell survival despite productive viral infection (Jones et al., 2014; Knights, 2017). Interestingly, the recent finding that CD8 T depletion could significantly enhance latency reversal indicates that CD8 T cells may block HIV-1 reactivation by LRAs (McBrien et al., 2020).

These findings emphasize the need for a more specific and potent “kill” function, such as LRAs that enhance the clearance of infected cells or combinations of treatment strategies to aid CTL function. To this end, TLR agonists offer promise due to their ability to induce a broad anti-viral response, simultaneously activating virus production and priming immune clearance of HIV-1 infected cells (Borducchi et al., 2016; Tsai et al., 2017; Lim et al., 2018; Macedo et al., 2018). To circumvent the need for CTL mediated cell clearance altogether, an alternative approach is to block the release of virions and induce apoptosis of the infected cell (Tateishi et al., 2017). In this method, a novel compound is used to inhibit HIV-1 Pr55^Gag^, blocking virus budding and leading to a build-up of viral products and subsequent apoptosis of the infected cell (Figure 1; Tateishi et al., 2017).

### Block and Lock

Recently, a novel cure strategy has been proposed that, rather than inducing latency reversal, aims to reinforce latency to prevent viral rebound following ART interruption (Figure 1; Mousseau et al., 2015; Méndez et al., 2018). The so called “block and lock” approach utilizes small interfering RNAs (siRNAs) to induce transcriptional gene silencing (TGS) by disrupting the regulation of chromatin structure, thereby preserving the epigenetic mechanisms that maintain HIV-1 latency (Suzuki et al., 2008; Ahlenstiel et al., 2015; Méndez et al., 2018). Alternatively, latency may be enforced by the targeted inhibition of the HIV-1 positive regulator, Tat, to lock the viral replication cycle at transcription (Mousseau et al., 2015). Whilst these approaches offer a conceptual alternative cure mechanism to “shock and kill,” their development is still in preliminary stages and is yet to be tested in human trials.

### Gene Editing

The rise to prominence of gene editing tools such as CRISPR-Cas9 and zinc-finger nucleases (ZFN) has led to increased hope of a HIV-1 cure by targeting various host or viral genes to induce host resistance, enforce viral latency or silence integrated provirus. Gene editing approaches have the advantage of highly specific gene targeting, so unlike LRAs, can produce the desired outcome without global physiological impact. Nevertheless, off-target effects have been observed in a number of studies and may affect the safety of these methods (Kimberland et al., 2018). So far, the potential of ZFN targeted editing of host CCR5, to induce partial genetic resistance to HIV-1, has been tested in a clinical trial (Tebas et al., 2014). Most research, however, has focused on the use of CRISPR-Cas9 for its relatively simple approach and a number of studies have demonstrated its use in CCR5 or CXCR4 gene editing to induce host cell resistance to HIV-1 (Wang et al., 2014, 2017; Xu et al., 2017). This approach may also be used to specifically knockout or attenuate the HIV-1 provirus, for example, by targeting the LTR to disrupt viral gene expression or excise the integrated genome (Ebina et al., 2013; Hu et al., 2014; Kaminski et al., 2016; Lebbink et al., 2017; Yin et al., 2017; Bella et al., 2018; Wang Q. et al., 2018). Alternatively, various positions of the latent provirus could be targeted by CRISPR-Cas9 to induce multiple non-homologous end joining (NHEJ) associated indels that deactivate the virus through frame shift mutation (Liao et al., 2015; Ueda et al., 2016; Wang et al., 2016; Ophinni et al., 2018). Additionally, recent work has shown that, in combination with a novel drug delivery system, CRISPR-Cas9 directed editing of proviral DNA could effectively eliminate HIV-1 infection in mouse models (Dash et al., 2019). This technology could feasibly be used to target myriad steps in the viral replication cycle, however, its major limitation is its delivery, requiring viral vectors or lipid compounds, as reviewed (Xiao et al., 2019). To achieve clinically significant effects, the majority, if not all of the LR will need to be affected, which is a major challenge considering the array of anatomical compartments which host a significant proportion of latently infected cells.

### Therapeutic Vaccination

Rebound viremia from latently infected cells is detectable within weeks of ART interruption, though the exact cellular and anatomical source of this rebound varies between patients (De Scheerder et al., 2019). Therefore, rather than targeting this elusive source, therapeutic vaccination aims to eliminate or significantly diminish rebound viremia by priming the host immune response to HIV-1, thereby achieving a “functional cure.” In therapeutic vaccine trials, the vaccine regimen is administered during sustained ART mediated viral suppression, followed by a period of ART interruption, during which vaccine efficacy can be assessed by measuring time to viral rebound, size of the LR and the profile of the host immune response.

Therapeutic vaccines may aim to elicit narrow CTL responses to specific HIV-1 proteins, such as Gag, though the success of these approaches may be impeded by the re-emergence of CTL escape mutants that were established during primary infection (Schooley et al., 2010; Pollard et al., 2014; Deng et al., 2015). Alternatively, vaccines designed to generate a broader anti-HIV-1 immune response may be more effective. To this end, several studies have used a dendritic cell (DC) based vaccine, in which autologous DCs are pulsed with inactivated HIV-1, or transfected to produce viral proteins, with the aim of generating DCs that can efficiently stimulate T cell responses (García et al., 2011; Gandhi et al., 2016; Gay et al., 2017b). Further, a vaccine that expresses multiple HIV-1 proteins may be used to induce a multivalent immune response, and previous studies combining such vaccines with IL-2 to boost T cell survival have demonstrated moderate success, with increased time to viral rebound associated with HIV-1 specific T cell responses in vaccinated participants (Lévy et al., 2005, 2006). Of note, a recent report has demonstrated continual decreases in the proviral reservoir as well as recovery of immune function following Tat based immunization, signifying that therapeutic vaccination can improve the immune response to HIV-1 (Sgadari et al., 2019).

Despite the promise of vaccine-based approaches, no study has yet induced sustained viral remission in vaccinated patients and in their recent analysis, Davenport et al. suggest that, even with highly efficacious vaccines that block 80% of viral reactivations, rebound viremia would likely emerge within 5 weeks following ART interruption (Davenport et al., 2019). This suggests that therapeutic vaccination alone may not be sufficient to cure HIV-1 infection and that instead, combinations of cure strategies may be more effective. For example, considering that “shock and kill” strategies have so far failed to achieve meaningful reduction in the LR, combining these strategies with therapeutic vaccination may increase the efficacy of each treatment. Indeed, this principle was tested in a clinical trial where Gag based vaccination was followed by HDCAi latency reversal and though this study was able to significantly reduce the LR, rebound viremia was measured within 2 weeks (Leth et al., 2016; Tapia et al., 2017).

### Novel Cure Strategies

Several novel approaches to induce sustained viral remission in treated patients have been proposed. One such method utilizes the relatively new discovery that exhausted CD4 T cells expressing immune checkpoint (IC) makers such as PD1 and CTLA-4, are a major reservoir of replication competent provirus (Banga et al., 2016; Fromentin et al., 2016; Castellano et al., 2017; McGary et al., 2017). IC markers are inhibitory receptors expressed by T cells in response to chronic viral infection to attenuate their effector function and limit tissue damage associated with long term immune activation (Boyer and Palmer, 2018). Cells expressing these markers, that are enriched in latent provirus, could therefore be specifically targeted for drug delivery or clearance using PD1, CTLA-4, or PD-L1 antibodies (Pantaleo and Levy, 2016; Gay et al., 2017a; Boyer and Palmer, 2018). To this end, several studies have demonstrated that IC blockade can inhibit the establishment of latency *in vitro* and aid latency reversal *in vivo*, revealing its potential as an HIV-1 therapeutic (McManamy et al., 2014; Gay et al., 2017a; Evans et al., 2018; Fromentin et al., 2019; van der Sluis et al., 2020).

Alternatively, following the success of chimeric antigen receptor T cells (CAR-T) in cancer therapy, their potential to treat HIV-1 is the subject of ongoing research. CAR-T cells are autologous T cells genetically engineered to express disease specific antibodies linked to an intracellular T cell receptor domain; therefore, when re-administered to the patient can direct the CTL response to cells expressing the disease epitope (Wagner, 2018). As such, this technology could be used to direct CTL mediated clearance of HIV-1 infected cells, aiding control of the virus in the absence of therapy. Currently, several studies using anti-HIV-1 CAR-T cells have demonstrated virus-clearing function *in vitro* (Sahu et al., 2013; Liu et al., 2015; Ali et al., 2016; Hale et al., 2017; Sung et al., 2018). More recently, a multi-specific CAR-T cell demonstrated potent clearance of HIV-1 infected cells in a humanized mouse model (Anthony-Gonda et al., 2019). The use of CAR-T cells is therefore an exciting new prospect in HIV-1 therapeutics and may work synergistically with LRAs to add more killing power into the “shock and kill” approach.

As discussed earlier, myeloid cells such as macrophages are known to support virus replication and may represent an additional barrier to HIV-1 cure. The use of “shock and kill” may not be effective against these cellular reservoir as they are refractory to CTL mediated immune clearance and the viral cytopathic effect (Swingler et al., 2007; Clayton et al., 2018). To address this, researchers have demonstrated differential expression of an anti-apoptotic, long non-coding RNA (lncRNA) that promotes survival of HIV-1 infected macrophages (Boliar et al., 2019). This study also showed that inhibition of this lncRNA with small interfering RNAs (siRNAs) could induce apoptosis in HIV-1 infected macrophages, indicating the potential of targeting lncRNAs as a novel therapeutic approach to aid the clearance of the LR in all cell types (Boliar et al., 2019).

## Assays to Measure the Success of HIV-1 Cure

### Viral Outgrowth Assay

**Figure 2:**
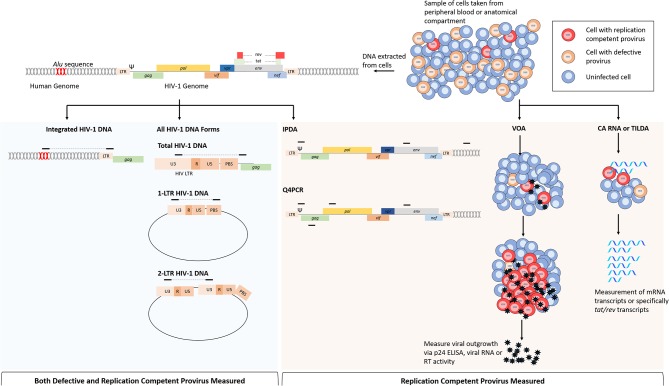
Comparison of assays that measure replication competent provirus specifically or all provirus. Cells for analysis come from either peripheral blood or from anatomical compartments. From left to right: following DNA extraction, multiple HIV-1 DNA forms can be assayed by PCR based on the primer position. For integrated HIV-1 DNA assays, a primer targeting repeated *Alu* sequences within the human genome are paired with a HIV-1 specific primer. Total HIV-1 DNA can be measured by primers specific for regions within the viral genome, this is most commonly performed with primers targeting conserved regions within the LTR. Non-integrated HIV-1 DNA forms such as 2-LTR and 1-LTR circular DNA can be measured by primers specific that will amplify junctions that are only present in these DNA forms. The intact proviral DNA assay (IPDA) uses primers within the packaging signal (Ψ) and *env* to determine replication competence. This assay also uses primers targeting regions within the human genome to measure cell numbers and correct for DNA shearing. Replication competence is determined when both sequences are present from ddPCR. The quadruplex PCR (Q4PCR) uses primers within Ψ, *env, gag*, and *pol* to quantify provirus in limiting dilutions, and NGS is uses to confirm replication competence in reactions with 2/4 of the sequences present. Cell based assays use purified cell samples to measure virus or RNA production following stimulation. The viral outgrowth assay (VOA) uses limiting dilutions of CD4 T cells that are stimulated with PMA and irradiated PBMCs to induce viral gene expression; viral outgrowth is supported by incubation with HIV-1 negative donor cells and measured by p24 ELISA, viral RNA or reverse transcriptase activity. Cell associated (CA) RNA or *tat/rev* induced limiting dilution assays measure viral RNAs following HIV-1 activation, reducing time to read out when compared to the VOA. Assays in blue shaded area are not specific for cells infected with replication competent provirus because viral DNA is measured indiscriminately. Assays in shaded orange area are more specific for replication competent provirus, or in the case of the VOA, only measure replication competent provirus.

Assessing the efficacy of HIV-1 cure and vaccine trials requires assays that reproducibly measure different virological markers to estimate the size of the LR with limited error. This is inherently challenging because of the relatively low abundance of latently infected cells and the heterogeneity of the HIV-1 genome, though several assays have been developed to this end (Table 1). Additionally, very few proviruses can generate infectious virions following activation and it is difficult to quantify specifically the replication competent reservoir. The standard assay used to measure intact provirus is the functional viral outgrowth assay (VOA) (Figure 2; Finzi et al., 1997, 1999; Siliciano and Siliciano, 2005). In this assay, limiting dilutions of CD4 T cells are stimulated to reverse latency and drive HIV-1 expression from integrated provirus. Activation of CD4 T cells is most commonly achieved via the addition of phytohemagglutinin (PHA) and CD8 T cell depleted PBMCs or by incubation with anti-CD28/CD3 antibodies (Wong et al., 1997; Finzi et al., 1999; Siliciano and Siliciano, 2005; Laird et al., 2013; Bruner et al., 2015). Following activation, viral outgrowth is supported by incubation with CD4 T cells from HIV-1 negative donors for 2–3 weeks and measured via the detection of p24 capsid antigen ELISA. Cell positive for exponential viral replication are quantified and the frequency of cells latently infected with intact provirus is determined based on Poisson distribution and expressed as infectious units per million (IUPM) cells (Siliciano and Siliciano, 2005; Rosenbloom et al., 2015).

**Table 1:** Different methods used to measure the latent reservoir.

**Assay**		**Advantages**	**Disadvantages**	**Examples**
Viral outgrowth assay (VOA)	Stimulated patient CD4 T cells in limiting dilution grown with donor cells and outgrowth measured	-Only measures replication competent provirus	-Time consuming -Requires large volumes of patient material -Underestimate size of the reservoir	Finzi et al., 1997 Siliciano and Siliciano, 2005 Laird et al., 2013 Bruner et al., 2015 Fun et al., 2017 Badia et al., 2018 Massanella et al., 2018 Wonderlich et al., 2019
Total HIV-1 DNA qPCR	Measures proviral DNA from cell extracts using primers/probes in conserved regions, primarily within the LTR	-Fast time from sample collection to result-Relatively inexpensive-Small sample volume-Can be used to detect different DNA forms (2-LTR, integrated)	-Cannot distinguish between intact and defective provirus so overestimates the reservoir -Quantification relative to a standard so prone to bias -Highly specific and prone to error from primer/template mismatches	Kostrikis et al., 2002 Beloukas et al., 2009 van der Sluis et al., 2013 Munir et al., 2013 Casabianca et al., 2014 Rouzioux et al., 2014 Vandergeeten et al., 2014 Thomas et al., 2019
Integrated HIV-1 DNA	Specifically measures only integrated provirus using a primer specific to HIV-1 and to *Alu* sequences randomly dispersed in the human genome	-Measures the LR by excluding unintegrated DNA forms-Fast and relatively inexpensive	-Distances between *Alu* and HIV-1 means ~10% of integrated provirus is measured -Heterogeneous nature of integration sites means standard design is complex	Brussel et al., 2005 Yu et al., 2008 Liszewski et al., 2009 Brady et al., 2013 Agosto et al., 2007 De Spiegelaere et al., 2014 Vandergeeten et al., 2014 Lada et al., 2018
Digital PCR	Measures frequency of proviral DNA (integrated, total or circular) by partitioning sample into limiting dilutions and assigning partitions either positive or negative	-Eliminates the need for a standard and so reduced bias (especially useful for integrated and 2-LTR circular DNA quantifications)	-More expensive and less widely available than standard qPCR methods -Suffers from false-positives inherent to the method -Setting thresholds to determine distinguish truly positive and negative partitions is difficult	De Spiegelaere et al., 2014 Henrich et al., 2012 Strain et al., 2013 Malatinkova et al., 2015 Henrich et al., 2017 Lada et al., 2018
Cell associated RNA	Measures all or different forms of cell associated RNA with the rationale that it is more likely to measure replication competent provirus than defective	-More sensitivity for replication competent provirus	-Cannot distinguish transcripts that arise from replication competent cells and defective cells	Archin et al., 2012 Pasternak et al., 2012 Shan et al., 2013 Cillo et al., 2014 Yucha et al., 2017 Massanella et al., 2018 Yukl et al., 2018
TILDA	Measures multiply spliced tat/rev transcripts following stimulation of CD4 T cells plated in limiting dilution	-Higher sensitivity for replication competent provirus-Faster, cheaper and less resources needed than VOA	Measured transcripts may arise from defective proviral genomes	Procopio et al., 2015 Frank et al., 2019 Bertoldi et al., 2020
ISH and flow cytometry	Measures mRNA and viral proteins measured following T cell activation	-Higher sensitivity for replication competent provirus-Simultaneously phenotype the cells that host the reservoir	-Does not confirm that RNA or proteins produced arise from replication competent provirus	Graf et al., 2013 Baxter et al., 2016, 2017 Martrus et al., 2016 Grau-Expósito et al., 2017 Deleage et al., 2018 Pardons et al., 2019a
IPDA	Multiplex digital PCR based assay to measure intact provirus based on the presence of two regions that are frequently mutated in the viral genome	-Enables distinction between intact and defective provirus-Faster readout than viral outgrowth assay	-Does not screen the whole genome and may therefore miss other deleterious mutations	Bruner et al., 2019
Q4PCR	Multiplex qPCR assay to assign replication competency based on presence of 4 genomic regions, confirmed by next generation sequencing if 2/4 are present	-Able to accurately distinguish intact and defective provirus-Filters out most defective provirus before using expensive sequencing	-Relatively expensive method	Gaebler et al., 2019

*The advantages and disadvantages of each approach as well as prominent examples*.

The original VOA provides high specificity for intact provirus but is limited by the large sample volume required, high resource cost and is susceptible to donor variation due to virus propagation in primary CD4 T cells (Bruner et al., 2015; Massanella and Richman, 2016). Several improvements of the VOA have attempted to overcome these limitations, including the use of continuous cell lines to improve reproducibility (Laird et al., 2013; Fun et al., 2017; Badia et al., 2018; Massanella et al., 2018) the use of RT PCR to detect HIV-1 RNA reducing time to read out (Laird et al., 2013) or utilizing improved p24 ELISA to increase sensitivity (Passaes et al., 2017). Recently, a novel improvement of the VOA has been described in which CD4 T cells are differentiated into effector cells to promote expression of HIV-1, enhancing cell activation and thereby increasing the sensitivity of the assay (Wonderlich et al., 2019). Additionally, an *in vivo* VOA, whereby humanized mouse models are used to support viral outgrowth, has been shown to increase sensitivity and detect virus replication in samples that were previously negative when quantified using traditional VOA (Metcalf Pate et al., 2015; Charlins et al., 2017).

The ability to distinguish intact and defective provirus has made the VOA assay the gold standard method to measure the LR, thought this assay underestimates the size of the intact LR by ~25 to 60-fold (Ho et al., 2013; Bruner et al., 2016). Genetic characterization of cells negative for viral outgrowth has revealed the presence of intact provirus, within active transcription units that is capable of generating replication competent virions following successive rounds of PHA stimulation (Ho et al., 2013; Hosmane et al., 2017). The mechanism underpinning the initial failure of these cells to generate viral outgrowth is likely the result of the stochastic nature of virus activation (Weinberger and Weinberger, 2013), nevertheless, their presence indicates an additional hurdle in both eradicating the LR and assessing the efficacy of eradication strategies. Of note, an extensive analysis of VOA performance using the same samples across different labs has indicated significant variability of results both within batches and between labs that is more pronounced in lower IUPM samples (Rosenbloom et al., 2019). This finding may have significant implications for HIV-1 cure research, where small differences in the replication competent reservoir must be accurately and reproducibly measured to assess the efficacy of therapeutic interventions.

### qPCR Based HIV-1 Quantification

Quantification of cell associated DNA by PCR provides a fast and relatively inexpensive marker to measure the size of the viral reservoir. HIV-1 DNA quantification methods rely on amplification of short genomic regions and so cannot distinguish intact and defective provirus and therefore vastly overestimate the size of the LR (Figure 2; Eriksson et al., 2013). Despite this limitation, HIV-1 DNA quantification has been shown to predict viral rebound (Williams et al., 2014) and offers the potential to identify different DNA forms, such as integrated HIV-1 DNA, non-integrated HIV-1 DNA (2-LTR and 1-LTR circular forms) or both (total HIV-1 DNA) (Mexas et al., 2012; Rouzioux and Avettand-Fenoël, 2018). Several factors affect the specificity, accuracy and reproducibly of HIV-1 DNA assays and as there is no standard method, meaningful comparison between different studies is limited. Currently, most HIV-1 DNA quantification assays utilize real-time quantitative PCR (qPCR) to measure the abundance of HIV-1 DNA relative to a calibration standard derived from cell lines harboring HIV-1 provirus. Cell lines such as 8E5 and ACH2 are widely used as the source of calibration DNA, though recent work has demonstrated that HIV-1 integration into these cell lines is unstable, likely due to ongoing replication, and their use may confound accurate quantification and reproducibility between labs (Sunshine et al., 2016; Wilburn et al., 2016; Busby et al., 2017; Symons et al., 2017; Rutsaert et al., 2018b; Thomas et al., 2019). Recent analysis of HIV-1 quantification methods has demonstrated the stability of HIV-1 integration into J-Lat 10.6, a Jurkat cell latently infected with full length, *env* deficient provirus, and suggested the use of this cell line as the gold standard for HIV-1 DNA quantification by qPCR (Sunshine et al., 2016; Thomas et al., 2019).

Another key determinant of the accuracy and specificity of HIV-1 DNA quantification assays is the genomic location at which the primers and probes anneal. The vast genetic variation of HIV-1 both within patients and across the epidemic necessitates appropriate selection of oligonucleotides that can efficiently amplify patient samples from a variety of sub-types and circulating recombinant forms (CRFs). Prominent assays have targeted various, highly conserved regions in the HIV-1 genome including *gag* (Kabamba-Mukadi et al., 2005; Kondo et al., 2009; Li et al., 2010) and *pol* (Désiré et al., 2001; Vitone et al., 2005). Nevertheless, the LTR region has been increasingly favored for HIV-1 DNA quantification because it is both highly conserved and facilitates the distinction between all of the various HIV-1 DNA forms (Kostrikis et al., 2002; Beloukas et al., 2009; Munir et al., 2013; van der Sluis et al., 2013; Casabianca et al., 2014; Rouzioux et al., 2014; Vandergeeten et al., 2014). Recently, an extensive *in silico* analysis of published HIV-1 DNA assays revealed substantial variation between different methods, especially when comparing quantification of different HIV-1 subtypes, and indicated the best performing assays for quantification of diverse patient cohorts (Rutsaert et al., 2018b).

As discussed above, LTR based DNA assays can distinguish different HIV-1 DNA forms. During HIV-1 replication, linear unintegrated cDNA accumulates in the cell as well as abortive DNA forms such as 1-LTR and 2-LTR circular DNA, which are products of recombination events and interaction with host DNA repair mechanisms (Sloan and Wainberg, 2011; Munir et al., 2013). Because 2-LTR circular forms arise from failed integration, they are considered markers of recent infection and their quantification may therefore provide insight into the replication competent reservoir (Buzón et al., 2010; Hatano et al., 2013; Kiselinova et al., 2016). Conflicting evidence, however, suggests that these DNA forms may be persistent for long periods in latently infected cells and the clinical relevance of 2-LTR quantification remains controversial (Pierson et al., 2002).

To exclude unintegrated DNA forms from quantification, it is possible to amplify specifically integrated provirus by targeting an endogenous *Alu* sequence that are found randomly across the human genome (Figure 2). Generally, *Alu* PCR assays utilize a nested approach in which the junction between an HIV-1 sequence and a human *Alu* sequence is amplified, followed by qPCR with primers specific to HIV-1 (Brussel et al., 2005; Agosto et al., 2007; Liszewski et al., 2009; Brady et al., 2013; De Spiegelaere et al., 2014; Vandergeeten et al., 2014; Ruggiero et al., 2017). *Alu* PCR remains the most common approach to measure integrated HIV-1 DNA, though alternative methods have been developed as reviewed here (Liszewski et al., 2009; Ruggiero et al., 2017). Whilst the *Alu* PCR assay has been shown to correlate well with the VOA (Eriksson et al., 2013), it is hindered by limitations in accuracy and sensitivity that are inherent to the method. The random dispersion of human *Alu* sequences, as well as the heterogeneity of HIV-1 integration sites, means that the sequence length between the *Alu* and HIV-1 specific primers is unknown and variable; presenting several technical challenges that may confound accurate quantification of proviral DNA (Brady et al., 2013). Cell lines used as quantification standards, for example, are generally derived from clonal, latently infected cells and therefore do not represent the random nature of integration within a patient sample (Ruggiero et al., 2017). To overcome this issue, researchers have developed a calibration standard containing multiple integration sites to resemble more closely the sample population (Agosto et al., 2007). Alternatively, the reliance on a standard may be circumvented by the use of repetitive sampling and absolute quantification based on Poisson distribution (De Spiegelaere et al., 2014). Additionally, only 10% of integrated HIV-1 is detected by this assay because 90% of integrated provirus is too far from an *Alu* sequence to be exponentially amplified and a correction factor must therefore be applied to the quantification (Agosto et al., 2007; Yu et al., 2008; Liszewski et al., 2009; De Spiegelaere et al., 2014). Accuracy is further limited by linear amplification of unintegrated HIV-1 DNA, though the effect of this can be partially negated by simultaneous pre-amplification with only the HIV-1 specific primer to enable distinction between integrated and unintegrated DNA (O'Doherty et al., 2002; Yu et al., 2008) or by pulsed-field gel electrophoresis (PFGE) prior to amplification to remove low molecular weight DNA (Lada et al., 2018). Despite its limitations and owing to the various improvements made, quantification integrated HIV-1 via *Alu* PCR is a powerful and high-throughput method to quantify the LR. An improved *Alu* PCR assay, where the HIV-1 LTR primer is closer to the integration junction and therefore detects more integration events, is currently in development (Personal Communication).

### Digital Droplet PCR Based HIV-1 Quantification

As discussed above, the selection of an appropriate calibration standard is required for quantification of HIV-1 DNA, however, quantification relative to a standard is inherently biased. Amplification efficiencies between the standard and the sample must be equal to limit bias when quantifying relative to a standard curve (Rutsaert et al., 2018a). Amplification efficiency is affected by the DNA input per reaction, the presence of inhibitory contaminants and, crucially for HIV-1 quantification, recent work has shown that small mismatches between the primer and target sequence significantly impair sample quantification (Rutsaert et al., 2018b; Thomas et al., 2019). Digital droplet PCR (ddPCR) platforms mitigate these issues by facilitating absolute quantification of a sample and as such, are becoming increasingly popular in HIV-1 research and clinical trials. In ddPCR, samples are randomly divided into multiple partitions and separately amplified, after which each partition is deemed positive or negative based on fluorescence above or below a threshold and absolute quantification is determined based on Poisson distribution (Hindson et al., 2011). In principle, the use of ddPCR to eliminate the need for a standard reduces these biases because each partition only needs to accumulate enough fluorescence to be deemed positive, so factors that reduce PCR efficiency should not impair the accuracy of quantification. The major limitation of ddPCR, however, is the difficulty to accurately determine the threshold above which a partition can be deemed positive (Rutsaert et al., 2018a). Partitions in which intermediate fluorescence is observed may be incorrectly assigned as positive or negative if the threshold selection is not sufficiently robust and a number of approaches to determine the threshold have been developed to overcome this issue, reviewed in detail here (Rutsaert et al., 2018a). Additionally, even with robust threshold selection, ddPCR is known to suffer from a high frequency of false-positive results (Henrich et al., 2012; Strain et al., 2013; Kiselinova et al., 2014; Bosman et al., 2015; Trypsteen et al., 2015). False-positives are likely the result of combined droplets resulting in increased fluorescence or from DNA contamination that is difficult to distinguish from truly positive samples (Henrich et al., 2012; Strain et al., 2013; Kiselinova et al., 2014; Bosman et al., 2015; Trypsteen et al., 2015). Despite these limitations, the use of ddPCR has proven an invaluable tool for measuring HIV-1 DNA and has been used successfully in various studies (De Spiegelaere et al., 2014; Malatinkova et al., 2015; Henrich et al., 2017).

### Bridging the Gap Between Culture and PCR Based Assays

Given that the majority of HIV-1 DNA is replication deficient, PCR based assays vastly overestimate the size of the latent reservoir (Eriksson et al., 2013; Ho et al., 2013). Conversely, the VOA is known to underestimate the size of the LR due to the presence of intact non-induced proviruses and so both methods may confound the assessment of treatment and cure strategies (Eriksson et al., 2013; Ho et al., 2013). Several assays have been developed with the aim to bridge the gap between these two types of analyses by providing a fast and relatively inexpensive method to specifically quantify only replication competent provirus. In a method conceptually similar to the VOA, cell associated (CA) HIV-1 RNA quantification following CD4 T cell activation has been used to measure the size of the inducible LR (Figure 2; Archin et al., 2012; Pasternak et al., 2012; Shan et al., 2013; Cillo et al., 2014; Yucha et al., 2017; Massanella et al., 2018; Yukl et al., 2018). The measurement of CA RNA provides the opportunity to quantify different transcripts and therefore, different stages of the replication cycle that may be used as a surrogate for measuring the size of the intact LR (Cillo et al., 2014; Massanella et al., 2018; Pasternak and Berkhout, 2018; Yukl et al., 2018). However, cells harboring defective provirus are still capable of producing HIV-1 mRNA following T cell activation despite being unable to generate infectious virions, and so these methods are prone to false positive results (Hermankova et al., 2003; Pasternak et al., 2009; Schmid et al., 2010; Cillo et al., 2014). By measuring cell-free HIV-1 RNA from culture supernatant, indicative of virus release from cells, as well as CA RNA, it is possible to more closely predict replication competence (Cillo et al., 2014; Massanella et al., 2018). In addition, a novel assay has addressed this issue by specifically measuring *tat/rev* multiply spliced mRNAs with the rationale that these transcripts are rarely produced in cells with defective HIV-1 provirus (Figure 2; Procopio et al., 2015; Frank et al., 2019; Bertoldi et al., 2020). The *tat/rev* induced limiting dilution assay (TILDA) relies on measurement of *tat/rev* transcripts from cells plated in limiting dilution, following activation with phorbol 12-myristate 13-acetate (PMA) and ionomycin (Procopio et al., 2015). Results obtained from TILDA quantification correlated well with HIV-1 DNA quantification and measures the LR close to levels predicted by Ho et al. (2013) and Procopio et al. (2015). This method, however, did not significantly correlate with results obtained from VOA and is still susceptible to overestimating the size of the LR due to the possibility that these transcripts arise from cells with defective HIV-1 genomes (Procopio et al., 2015).

Other groups have sought to quantify the replication competent reservoir using *in situ* hybridization (ISH) and flow cytometry to measure CA RNA or capsid p24 protein (Graf et al., 2013; Baxter et al., 2016, 2017; Martrus et al., 2016; Grau-Expósito et al., 2017; Deleage et al., 2018; Pardons et al., 2019a). By combining flow cytometry based quantification of CA RNA and p24 capsid protein, it is possible to measure provirus that is capable of transcription as well as protein production, providing a close surrogate for the measurement of the intact LR (Baxter et al., 2016, 2017, 2018; Martrus et al., 2016; Grau-Expósito et al., 2017; Puray-Chavez et al., 2017). An additional benefit of flow cytometry based approaches is the opportunity to simultaneously infer phenotypic characteristics of the cell populations that host the replication competent reservoir, as reviewed (Baxter et al., 2018).

More recently, a novel assay known as the intact proviral DNA assay (IPDA) has demonstrated the use of a multiplexed ddPCR approach to measure the size of the intact LR based on the presence of regions that are frequently mutated in defective genomes (Figure 2; Bruner et al., 2019). In this assay, intact and defective proviruses are separately quantified by amplifying regions within the HIV-1 packaging signal (Ψ) and *env* and the presence or absence of these regions is sufficient to distinguish 90% of defective genomes (Bruner et al., 2019). By determining replication competence based on DNA composition, this assay is not dependent on T cell stimulation and is therefore not impaired by the presence of non-inducible, intact proviruses that contribute to LR underestimation in the VOA (Bruner et al., 2015, 2016). Despite this, the IPDA is still only able to distinguish 90% of defective proviruses, with mutations that occur in non-amplified regions counting toward the quantification. Additionally, like all PCR based HIV-1 assays, primer mismatches in target regions may result in false negative quantifications. Similarly, Gaebler et al., recently described an approach (Q4PCR) that uses multiplexed qPCR measurement of four proviral regions; *gag, pol, env*, and Ψ, followed by next generation sequencing (NGS) of samples that are positive for two out of four regions to confirm replication competence (Figure 2; Gaebler et al., 2019). In comparison with IPDA, the Q4PCR method offers increased accuracy to predict replication competence due to a higher percentage of the viral genome being interrogated and likely positive samples being validated via NGS (Gaebler et al., 2019). Nevertheless, this increased sensitivity does come with the increased cost and lower throughput associated with NGS.

Previously, full-length sequencing of proviral DNA has provided invaluable insight into the composition of the LR (Ho et al., 2013) but the methods used are time consuming and technically challenging. The advent of various NGS technologies, however, has also paved the way for novel methods to measure the HIV-1 LR with relative ease and high throughput (Lambrechts et al., 2020). The use of Illumina based sequencing techniques has so far been used in LR studies to measure full-length, individual proviral sequences, helping to elucidate the driving force of LR persistence and latency maintenance (Hiener et al., 2017; Lee et al., 2017; Einkauf et al., 2019). Further, the emergence of NGS technologies that can sequence long-reads, such as PacBio's SMRT Sequencing and Oxford Nanopore's MinION, may be employed to measure full-length proviral genomes or variant transcript forms from patient samples and are likely to lead to advances in our understanding of the LR.

## Conclusions

The use of antiretroviral therapy has succeeded in reducing HIV-1 mortality but cannot eliminate the virus due to the persistent and stable LR. The global disease burden, equating to ~36 million infected individuals of which ~22 million have access to ART, warrants the continued search for a therapeutic approach that can either eliminate the virus or induce sustained viral remission in the absence of therapy (Sung et al., 2018). Recent advances in our understanding of the LR, its cellular and anatomical hosts and the mechanisms that facilitate its long-term persistence have contributed to renewed hope of a curative intervention for HIV-1 infection. Generally, an HIV-1 cure should eliminate the possibility of viral rebound following treatment interruption, and this relies on drastic reduction in the LR and efficient immune mediated clearance of HIV-1 infected cells.

Currently, several approaches for HIV-1 cure have been proposed and trialed to varying degrees of success. One of the most prominent cure strategies, “shock and kill,” has demonstrated virus reactivation *in vivo*, but has been unable to lead to a meaningful increase in the time to viral rebound; suggesting improvement is required to aid the “killing” of infected cells. Alternative approaches, such as therapeutic vaccination, aim to prime the immune response to HIV-1 infection with the rationale that upon treatment interruption, immune mediated control of the virus will be improved. Several new technologies and approaches, such as immune checkpoint inhibitors, gene editing and CAR-T cells may offer an alternative method for cure, though currently their assessment in clinical trials is limited. An added complication in the search for an HIV-1 cure is the difficulty in accurately measuring the success of such trials. The inherent variability of the HIV-1 genome, the low frequency of latently infected cells as well as the abundance of defective provirus contribute to the complexity of LR quantification.

Rather than an improvement in the current strategies leading to a cure, it is likely that synergistic combinations of different approaches, such as the use of LRAs following therapeutic vaccination, will lead to more drastic reductions in the LR and may aid the ultimate goal of long term ART free viral remission.

## Author Contributions

JT conceptualized and outlined the manuscript and wrote the first draft. AR, WP, and GP contributed to editing the manuscript. All authors approved the final version.

### Conflict of Interest

The authors declare that the research was conducted in the absence of any commercial or financial relationships that could be construed as a potential conflict of interest. The handling editor declared a past co-authorship with one of the authors GP.
